# Supplementary material for: Unblended disjoint tree merging using GTM improves species tree estimation
Source: BMC Genomics. 2020 Apr 16;21(Suppl 2):235. doi: 10.1186/s12864-020-6605-1 (PMC7161100; doi:10.1186/s12864-020-6605-1)
Supplement: Supplementary file 1 — Additional file 1 Codes used in the performance study. This document provides the commands used to perform the simulation study. [file 12864_2020_6605_MOESM1_ESM.pdf]

Additional File 1: Codes used in the performance study

Vladimir Smirnov Tandy Warnow

August 15, 2019

1 Codes for computing trees

ASTRAL 5.6.3
Code available at [1].
Command:
```
java -jar astral.5.6.3.jar
-i gene_trees.tre -o output_tree.tre
```

ASTRID 1.4
Code available at [2]. Command:
```
ASTRID-linux -i gene_trees.tre
-o output_tree.tre -c output_matrix.mat
```

FastTree 2.1
We ran FastTree in an unpartitioned analysis (i.e., treating all the loci as evolving down the same model tree).
Command:
```
FastTree -nt -gtr
alignment.fas > output_tree.tre
```

NJMerge
Code available at [3].
Command:
```
python3 njmerge.py
-t constraint_tree1.tre constraint_tree2.tre
-m distance_matrix.mat -o output_tree.tre
```

NJst
To compute the NJst tree, we use ASTRID (with the "-c" flag) to compute the matrix of average (across all gene trees) topological distances between species. We then compute a neighbor joining [4] tree using FastME v. 2.15 [5] with command:
```
fastMEPath -mN
-i distance_matrix.mat -o output_tree.tre
```

RAxML 8.2.10
We ran RAxML in an unpartitioned analysis (i.e., treating all the loci as evolving down the same model tree), and with only one random starting condition.
Command:
```
raxmlHPC-PTHREADS-SSE3
-m GTRGAMMA -F -p 12345
-n output -s alignment.fas -T 4
```

TreeMerge
Code available at [6].
Command:
```
python3 treemerge.py
-s start_tree.tre
-t constraint_tree1.tre constraint_tree2.tre
-m distance_matrix.mat -o output_tree.tre
-p paup4a165_centos64 -w workingDir
```

2 Other details

Obtaining a decomposition (1 round)

We used the script build_subsets_from_tree.py, found in tools.zip in the NJMerge data repository [7]. The method was executed by calling the decompose_trees function in the script with the tree object to decompose and a maximum subset size (120). However, the script can also be run through its main method by specifying the maximum subset size and the input tree file.

Applying ASTRAL to subsets of species

To construct ASTRAL trees on subsets, we first restrict each of the gene trees to the specified subset, then we run ASTRAL on the set of restricted gene trees. To restrict each gene tree to the relevant subset, we use the Dendropy [8] command `retain_taxa_with_labels(<subset taxa>)`. The resulting restricted gene trees are written file using Dendropy's as_string(schema="newick") function, and ASTRAL is called with that file as input.

Computing RF distance

To compute the RF distance between two trees, each on N leaves, we ran the compare_trees.py script, found in tools.zip in the NJMerge data repository [7], using the following command:

```
compare_trees.py <tree1> <tree2>
```

To return the RF error rate, we divided these values by $2N - 6$.

References

[1] Mirarab, S.: Github site for ASTRAL. https://github.com/smirarab/ASTRAL, last accessed August 15, 2019 (2019)

[2] Vachaspati, P.: Github site for ASTRID. https://github.com/pranjalv123/ASTRID-1, last accessed August 15, 2019 (2019)

[3] Molloy, E.K.: Github site for NJMerge. https://github.com/ekmolloy/njmerge, last accessed August 15, 2019 (2019)

[4] Saitou, N., Nei, M.: The neighbor-joining method: a new method for reconstructing phylogenetic trees. Molecular Biology and Evolution **4**(4), 406–425 (1987). doi:10.1093/oxfordjournals.molbev.a040454

[5] Lefort, V., Desper, R., Gascuel, O.: FastME 2.0: A Comprehensive, Accurate, and Fast Distance-Based Phylogeny Inference Program. Molecular Biology and Evolution **32**(10), 2798–2800 (2015). doi:10.1093/molbev/msv150

[6] Molloy, E.K.: Github site for TreeMerge. https://github.com/ekmolloy/treemerge, last accessed August 15, 2019 (2019)

[7] Molloy, E.K.: Illinois Data Bank repository for the NJMerge paper. https://databank.illinois.edu/datasets/IDB-1424746, last accessed August 15, 2019 (2019)

[8] Sukumaran, J., Holder, M.T.: DendroPy: a Python library for phylogenetic computing. Bioinformatics **26**(12), 1569–1571 (2010). doi:10.1093/bioinformatics/btq228
