# Supplementary material for: Unblended disjoint tree merging using GTM improves species tree estimation
Source: BMC Genomics. 2020 Apr 16;21(Suppl 2):235. doi: 10.1186/s12864-020-6605-1 (PMC7161100; doi:10.1186/s12864-020-6605-1)
Supplement: Supplementary file 2 — Additional file 2 Additional proofs. This document provides proofs of Theorems 1–3. [file 12864_2020_6605_MOESM2_ESM.pdf]

Additional File 2: Additional Proofs

Vladimir Smirnov   Tandy Warnow

March 3, 2020

1 Proof of Theorem 1

Recall that the input to the DTM-GT-FN problem is a set $\mathcal{T}$ of unrooted leaf-disjoint trees, with leaves labelled by species in S, and a guide tree T^* with leaves labelled from S, and the objective is to find a tree T' that has the minimum FN distance to T and that is achieved by adding edges to connect the trees in $\mathcal{T}$.

Theorem 1 DTM-GT-FN is NP-hard

Proof. Consider the decision problem **DTM-GT-FN-0**, which is given the same inputs as DTM-GT-FN and asks "is there a tree T' with 0 FN distance from the guide tree T^*?" We will show that DTM-GT-FN-0 is NP-hard, from which the NP-hardness of DTM-GT-FN naturally follows.

We reduce the Unrooted Tree Compatibility problem to DTM-GT-FN-0. The Unrooted Tree Compatibility decision problem is given a set of unrooted trees on overlapping leaf sets, determine if there exists a tree that induces them all. Unrooted Tree Compatibility is known to be NP-complete [1] (see also discussion in [2]), so this reduction will establish the NP-hardness of DTM-GT-FN-0. Let $T_1, T_2, \ldots T_k$ be unrooted binary trees on overlapping subsets of leaf set L. The following polynomial-time procedure will convert these inputs to an instance of DTM-GT-FN-0:

- First, we define a new leaf set L' as follows: for each leaf $l \in L$ that appears in n trees $T_{a_1}, T_{a_2}, \ldots T_{a_n}$, L' will contain n leaves $l_{a_1}, l_{a_2}, \ldots l_{a_n}$.

- Second, we produce a set of disjoint constraint trees on L': for each tree T_i, we produce a constraint tree T_i' by replacing each leaf $l \in L$ in T_i with leaf $l_i \in L'$. Since each leaf in each constraint tree is indexed by the tree it's in, the trees are trivially disjoint.

- Third, we produce our guide tree T^*: we include the trivial bipartition for each leaf in L', and, for each leaf $l \in L$ that corresponds to $l_{a_1}, l_{a_2}, \ldots, l_{a_n} \in L'$, T^* gets one bipartition separating $l_{a_1}, l_{a_2}, \ldots, l_{a_n}$ from all other leaves: $(l_{a_1} l_{a_2}, \ldots, l_{a_n} | L' - \{l_{a_1}, l_{a_2}, \ldots, l_{a_n}\})$. Topologically, we can build such a tree by starting with a star tree on the leaves

in L, and replacing each leaf l with an internal node, whose children are the corresponding leaves $l_{a_1}, l_{a_2}, \ldots, l_{a_n}$. Note that the construction of the constraint trees and the guide tree takes polynomial time and their size is bounded by a polynomial in the input to Unrooted Tree Compatibility. To complete the proof, we need to verify that solving DTM-GT-FN-0 on our guide tree T^* and our new constraint trees T_i' yields "yes" if and only if Unrooted Tree Compatibility yields "yes" on T_i.

If there exists some supertree A_1 that induces all trees T_i, then there exists a tree A_2 with 0 FN distance from T^*: we obtain A_2 from A_1 in the same fashion as we produced T^* earlier–by replacing each leaf $l \in L$ in A_1 with one internal node whose children are the corresponding $l_{a_1}, l_{a_2}, ...l_{a_n} \in L'$. Trivially, A_2 would contain all bipartitions from T^*, and, since A_1 induces each T_i, A_2 would likewise induce T_i'. On the other hand, if such an A_1 doesn't exist, then there is no A_2 with 0 FN distance from T^*: if such an A_2 exists, we could recover A_1 from A_2 by performing the opposite operation. Thus, the Unrooted Tree Compatibility problem reduces to DTM-GT-FN-0, showing that DTM-GT-FN-0 is NP-hard, and, therefore, DTM-GT-FN is NP-hard. □

2 Proof of Theorem 2

Theorem 2 Given a guide tree T^* and set $\mathcal{T} = \{T_1, T_2, \ldots, T_k\}$ of constraint trees over their respective leaf sets $L(T_i)$ of T_i^*, GTM optimally solves Unblended DTM-GT-FN on $(\mathcal{T}, T^*)$ and has a worst case running time of $O(N^2)$ where $N = |L(T^*)|$ is the total number of species.

Proof. We begin with proving correctness (i.e., that GTM finds an optimal solution to DTM-GT-FN), and then prove the running time.

(1) Proof of correctness: It is clear by construction that GTM produces an unblended compatibility supertree for $\mathcal{T}$. The only issue therefore is whether it returns a tree that has the smallest FN rate of all such supertrees. Then, given the recursive structure described above, it will suffice to show that each output of $Rejoin(T)$ satisfies all constraint trees T_i such that $L(T_i) \subseteq L(T)$ and contains all bipartitions in T. Recall that we collapsed all violating edges in T^*, as well as any edges that violate convexity. These two types of edges obviously may not appear in any valid "unblended joining" result, so if $Rejoin(T)$ contains all bipartitions of T, it achieves the best possible FN distance to $T^*|L(T)$.

The base case is when $L(T)$ corresponds to some $L(T_i)$, and $Rejoin(T)$ just returns the constraint tree, T_i. This case is trivially correct. Then, we can look at the inductive step, which is as follows. We divide T into S_1 and S_2 on bridge edge e. Our inductive hypothesis is that $S_1' = Rejoin(S_1)$ and $S_2' = Rejoin(S_2)$ satisfy all constraint trees on their respective leaf sets and contain all bipartitions of S_1 and S_2. We find attachment edges $a_1 \in S_1'$ and $a_2 \in S_2'$ from $e_1 \in S_1$ and $e_2 \in S_2$ as described in the procedure, join them

with edge e' to form T', and want to prove that T' satisfies all constraint trees $T_i : L(T_i) \subseteq L(T)$ and contains all bipartitions of T.

We first notice that an attachment edge $a_1 \in S_1$, such that $b(a_1) = b(e_1)$, must always exist: since $e_1 \in S_1$, our inductive hypothesis says that $b(e_1)$ must appear in S'_1. The same goes for a_2. Since S'_1 and S'_2 satisfy all constraint trees on their respective leaf sets and are connected by a single edge without modification to either one, then T' also straightforwardly satisfies both sets of constraint trees. Now, let s be any edge in T. We want to show that $b(s)$ must appear in T'. There are three possibilities:

1) s is an edge in S_1.

2) s is an edge in S_2.

3) s is e.

The third case is clear, since e and e' are the same bipartition $L(S_1)|L(S_2)$. WLOG, let's assume the first case. This means that $b(s)$ is a bipartition of the form $A|(B \cup L(S_2))$, where $A \cup B = L(S_1)$. Then, bipartition $A|B$ appears in S_1 and, by our inductive hypothesis, there's an edge $s' \in S'_1$, such that $b(s') = A|B$. When we attach e' to S'_1, $b(s')$ will become either $A|(B \cup L(S_2))$ or $(A \cup L(S_2))|B$, depending on which side of a_1 s' is on.

Let $b(e_1) = b(a_1) = A_a|B_a$, so, when e' is attached, edge a_1 splits into two bipartitions: $A_a|(B_a \cup L(S_2))$ and $(A_a \cup L(S_2))|B_a$. If $s = e_1$, then $b(s)$ is one of these. If $s \neq e_1$, we know that $B_a \subset B$, because otherwise, s would lie between e_1 and e, which is impossible by construction. Since $b(s')$ must be compatible with $A_a|(B_a \cup L(S_2))$, this forces $b(s')$ to be $A|(B \cup L(S_2))$. Otherwise, neither side of $(A \cup L(S_2))|B$ is a subset of either side of $A_a|(B_a \cup L(S_2))$, making them incompatible. Thus, $b(s)$ appears in T'.

(2) Proof that GTM has $O(N^2)$ worst case running time, where N is the number of taxa. The implementation of the GTM procedure can be summarized as shown in the algorithm block below.

This permits a fairly straightforward runtime analysis. The first step is the collapse of violating edges in T^*. In the interest of speed, we can facilitate this with a preprocessing step: for each constraint tree T_i, we encode its bipartitions as binary bitmasks (in the conventional way) and store these numerical values in a hash table H_{T_i}. Then, for each edge $e \in T^*$ with associated bitmask b, we can check which constraint trees e satisfies by hashing the bits of b associated with each constraint tree and seeing if this hash appears in H_{T_i}. This entails hashing $|L(T_i)|$ bits, which is $O(|L(T_i)|)$, followed by a lookup in a hash table with $O(|L(T_i)|)$ elements, which is also $O(|L(T_i)|)$ in the worst case. Checking all constraint trees, every edge performs an $O(\sum_i |L(T_i)|) = O(N)$ operation, and doing this for each of N edges in T^* makes this first part $\mathcal{O}(N^2)$. While we're traversing each edge, we also add some annotations to facilitate the second part of the algorithm quickly: based on which constraint tree each bit in b is associated with, we identify if e is a border edge separating which constraint trees, or which constraint set e lies in. This piggybacks on the total $O(N)$ cost for e.

Algorithm 1 GTM

for edge x in T^* **do**
    if x violates a constraint or violates convexity **then** collapse x
return Rejoin(T^*)

procedure REJOIN(T)
    for each T_i **do**
        if $L(T) = L(T_i)$ **then** return T_i
    $v \leftarrow$ border node
    $e \leftarrow$ get bridge edge from v
    $S_1, S_2 \leftarrow$ delete e from T
    $e_1 \in S_1, e_2 \in S_2 \leftarrow$ neighbors of e
    $S_1', S_2' \leftarrow Rejoin(S_1), Rejoin(S_2)$.
    for edge x in S_1' **do**
        if $b(x) = b(e_1)$ **then** $a_1 \leftarrow x$
    for edge x in S_2' **do**
        if $b(x) = b(e_2)$ **then** $a_2 \leftarrow x$
    $T' \leftarrow$ add edge between a_1 and a_2
    return T'

The second part is the recursive Rejoin itself. The recursion is invoked twice for each bridge edge, of which there are $\mathcal{O}(k)$, giving us $\mathcal{O}(k)$ calls to Rejoin. The check $L(T) = L(T_i)$ is $O(k)$, since it uses the annotations we computed in the previous part. (We know which constraint trees were separated by the edge we just deleted to produce T). The next tasks are to find a border node v, as well as a_1 and a_2, and these are $\mathcal{O}(N)$ operations likewise facilitated by the computations from the previous part. Everything else in the procedure is a unit-time operation. Thus, we end up with $\mathcal{O}(k)$ calls of a $\mathcal{O}(N)$ function, so the second part is $\mathcal{O}(Nk)$. Putting everything together, the entire procedure has worst case running time $\mathcal{O}(N^2)$. □

3 Proof of Theorem 3

Theorem 3: Let Φ be a model of evolution. If the method X used to construct the starting tree and the method Y used to construct the subset trees are both statistically consistent under Φ, then the DTM pipeline X-Y-GTM is also statistically consistent under Φ.

Proof. Let T be the model tree. Since X is statistically consistent, the starting tree produced by X will converge to the model tree, T, as the amount of data increases. When the starting tree is the model tree, the decomposition strategy (which is based on edge deletions from the starting tree) will produce subsets that are convex within the model tree. Since Y is also statistically consistent, the computed subset trees will also converge to the model species tree, restricted

to each subset. Given correct subset trees, each of which is based on a convex subset within the model tree, the optimal unblended DTM-GT-FN tree is the model tree. Since GTM optimally solves unblended DTM-GT-FN, it will return T when given such an input. Hence, as the amount of data increases, X-Y-GTM will converge to the model tree T. □

References

[1] Steel, M.A.: The complexity of reconstructing trees from qualitative characters and subtrees. Journal of Classification **9**, 91–116 (1992)

[2] Warnow, T.: Computational Phylogenetics: An Introduction to Designing Methods for Phylogeny Estimation. Cambridge University Press, Cambridge (2018)
