# Supplementary material for: Unblended disjoint tree merging using GTM improves species tree estimation
Source: BMC Genomics. 2020 Apr 16;21(Suppl 2):235. doi: 10.1186/s12864-020-6605-1 (PMC7161100; doi:10.1186/s12864-020-6605-1)
Supplement: Supplementary file 3 — Additional file 3 Additional figures. This document provides additional figures for the performance study. [file 12864_2020_6605_MOESM3_ESM.pdf]

Additional File 3: Additional Figures

Vladimir Smirnov Tandy Warnow

August 15, 2019

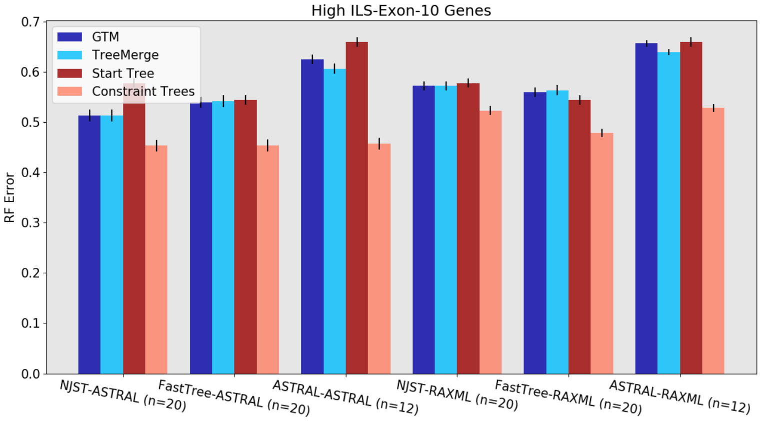

Figure S1: **Experiment 1: Tree error rates for GTM and TreeMerge with different "starting tree-constraint tree" combinations over 10 high ILS exon genes with 1000 species.** (NJMerge is not shown, because it failed for all high-ILS 10 and 25-gene replicates, see Figure S3 for high-ILS NJMerge results on 1000 introns). Both DTM methods work best with ASTRAL constraint trees and show a distinct preference for the NJst-ASTRAL combination. The value for n is the number of replicates being compared, where all methods ran. Error bars show standard error of the replicate average.

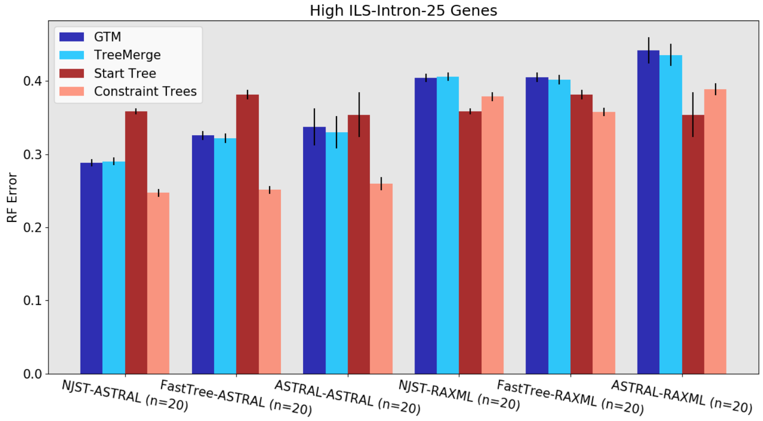

Figure S2: **Experiment 1: Tree error rates for GTM and TreeMerge with different "starting tree-constraint tree" combinations over 25 high ILS intron genes with 1000 species.** (NJMerge is not shown, because it failed for all high-ILS 10 and 25-gene replicates, see Figure S3 for high-ILS NJMerge results on 1000 introns). Both DTM methods work best with ASTRAL constraint trees and show a distinct preference for the NJst-ASTRAL combination. The value for n is the number of replicates being compared, where all methods ran. Error bars show standard error of the replicate average.

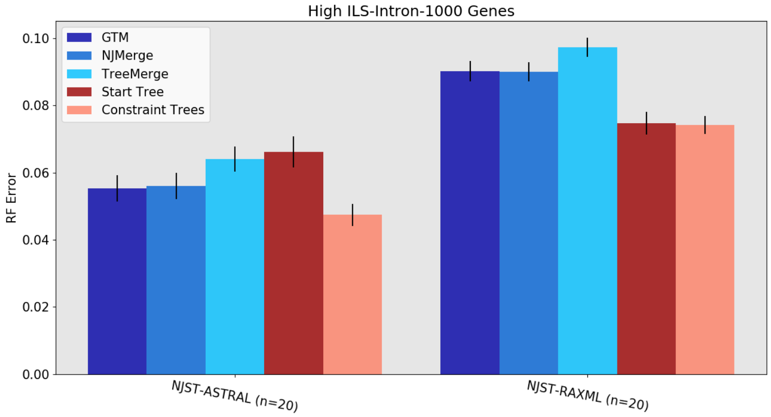

Figure S3: **Experiment 1: Tree error rates for GTM, NJMerge, and TreeMerge with NJst starting trees and ASTRAL/RAxML constraint trees over 1000 high ILS intron genes with 1000 species.** Only NJst starting trees are used, due to impracticability of FastTree and ASTRAL starting trees at this scale. All three DTM methods work best with ASTRAL constraint trees. The value for n is the number of replicates being compared, where all methods ran. Error bars show standard error of the replicate average.

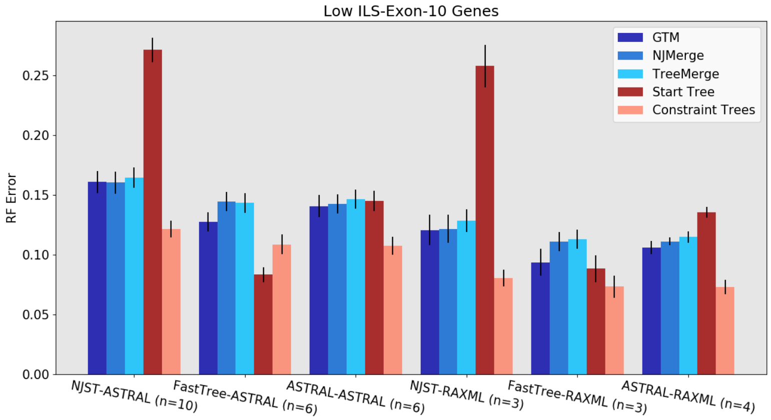

Figure S4: **Experiment 1: Tree error rates for GTM, NJMerge, and TreeMerge with different "starting tree-constraint tree" combinations over 10 low ILS exon genes with 1000 species.** All three DTM methods work best with FastTree starting trees and RAxML constraint trees. The value for n reflects the the number of replicates being compared, where all methods ran. Error bars show standard error of the replicate average.

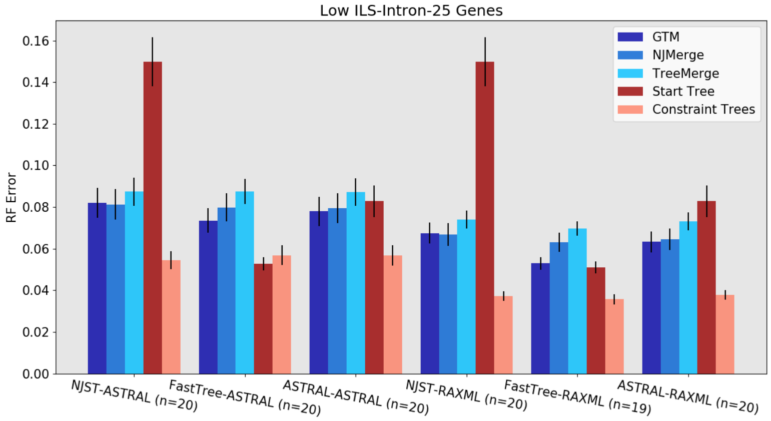

Figure S5: **Experiment 1: Tree error rates for GTM, NJMerge, and TreeMerge with different "starting tree-constraint tree" combinations over 25 low ILS intron genes with 1000 species.** All three DTM methods work best with FastTree starting trees and RAxML constraint trees. The value for n is the number of replicates being compared, where all methods ran. Error bars show standard error of the replicate average.

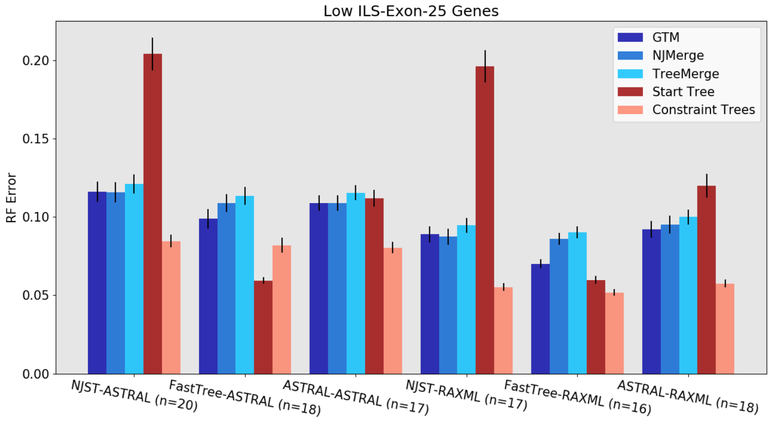

Figure S6: **Experiment 1: Tree error rates for GTM, NJMerge, and TreeMerge with different "starting tree-constraint tree" combinations over 25 low ILS exon genes with 1000 species.** All three DTM methods work best with RAxML constraint trees. All three methods improve with the FastTree starting tree, with a big improvement for GTM. The value for n is the number of replicates being compared, where all methods ran. Error bars show standard error of the replicate average.

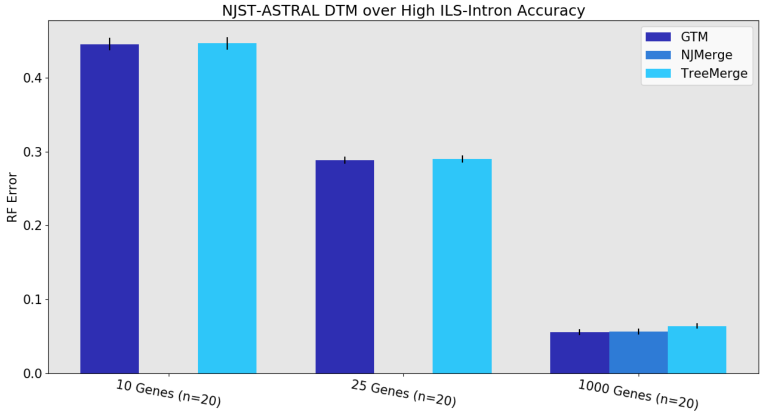

Figure S7: **Experiment 2: Tree error rates for three DTM methods on 1000 species given NJst starting tree and ASTRAL constraint trees, the best pipeline for high ILS from Experiment 1.** NJMerge failed to complete within the allowed 4 hour period for all 10- and 25-gene high ILS replicates (results shown for NJMerge on 1000-gene datasets are from [1]). GTM and TreeMerge are about even, with a slight advantage to GTM at 1000 genes. The value for n is the number of replicates being compared where all methods ran. Error bars show standard error of the replicate average.

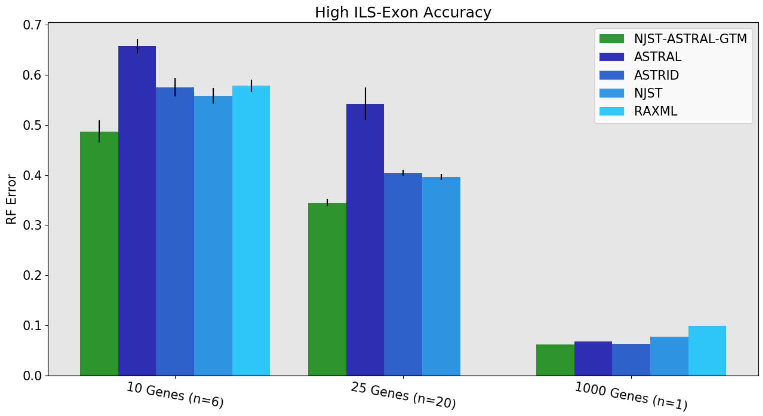

Figure S8: **Experiment 3: Tree error rates for NJst-ASTRAL-GTM, RAxML, and leading summary methods (ASTRAL, ASTRID, NJst) on high ILS datasets with 1000 species.** GTM is more accurate than the other methods on small numbers of genes, and roughly matches ASTRAL and ASTRID on 1000. The value for n is the number of replicates being compared, where all methods ran (i.e., where ASTRAL and RAxML are both available). RAxML timed out on 8 of the 10-gene replicates and all 25-gene replicates. ASTRAL timed out on 7 10-gene replicates. The 1000-gene ASTRAL and RAxML trees were taken from the NJMerge study [1], and only one ASTRAL tree is available for high ILS exons. Error bars indicate standard error of the replicate average.

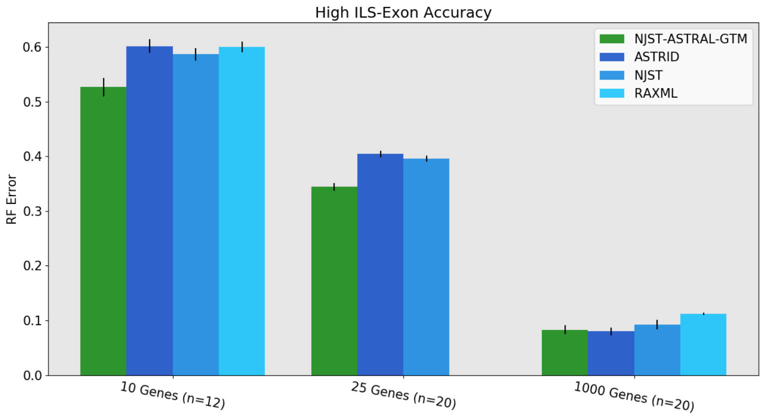

Figure S9: **Experiment 3: Tree error rates for NJst-ASTRAL-GTM, RAxML, and summary methods (ASTRID and NJst) on high ILS datasets with 1000 species.** NJst-ASTRAL-GTM is more accurate than the other methods on small numbers of genes. For 1000-gene datasets, NJst-ASTRAL-GTM and ASTRID are tied, and are more accurate than NJst and RAxML. The value for n is the number of replicates where RAxML trees are available. RAxML timed out on 8 of the 10-gene replicates and all 25-gene replicates. The 1000-gene RAxML trees were taken from the NJMerge study [1]. Error bars show standard error of the replicate average.

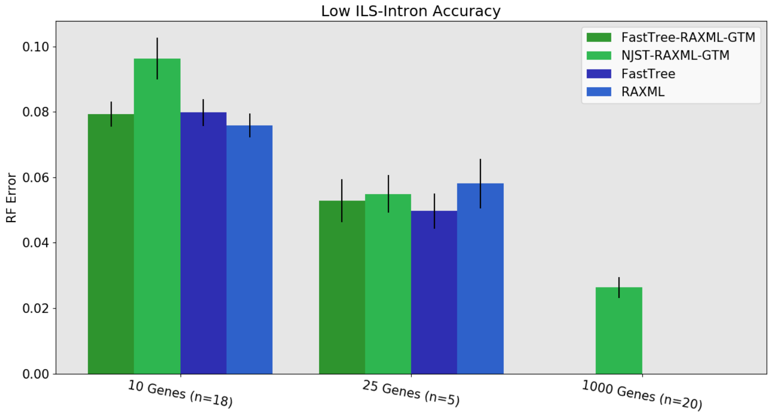

Figure S10: **Experiment 3: Comparison of FastTree-RAxML-GTM, NJst-RAxML-GTM, FastTree, and RAxML on 1000-species datasets with low ILS introns.** The value for n is the number of replicates on which RAxML completed; missing replicates indicate RAxML exceeding runtime limits on 10 and 25 genes, and RAxML trees were not available for 1000 genes. FastTree was not used for 1000 genes. Error bars show standard error of the replicate average.

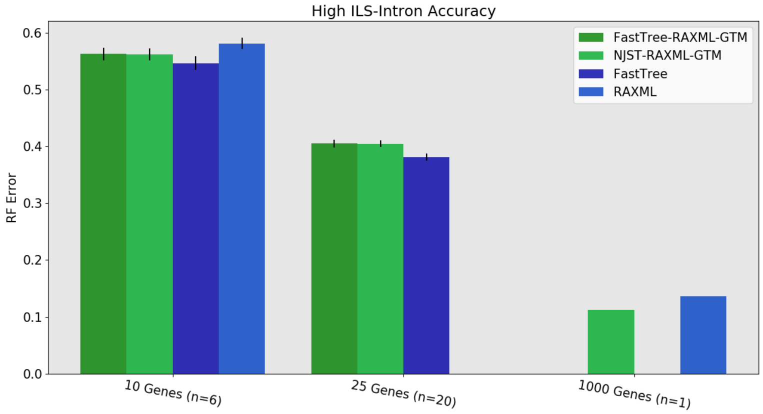

Figure S11: **Experiment 3: Comparison of FastTree-RAxML-GTM, NJst-RAxML-GTM, FastTree, and RAxML on 1000-species datasets with high ILS introns.** The value for n is the number of replicates on which RAxML completed; missing replicates indicate RAxML exceeding runtime limits on 10 and 25 genes (the 1000-gene RAxML trees are taken from [1]). FastTree was not used for 1000 genes. Error bars show standard error of the replicate average.

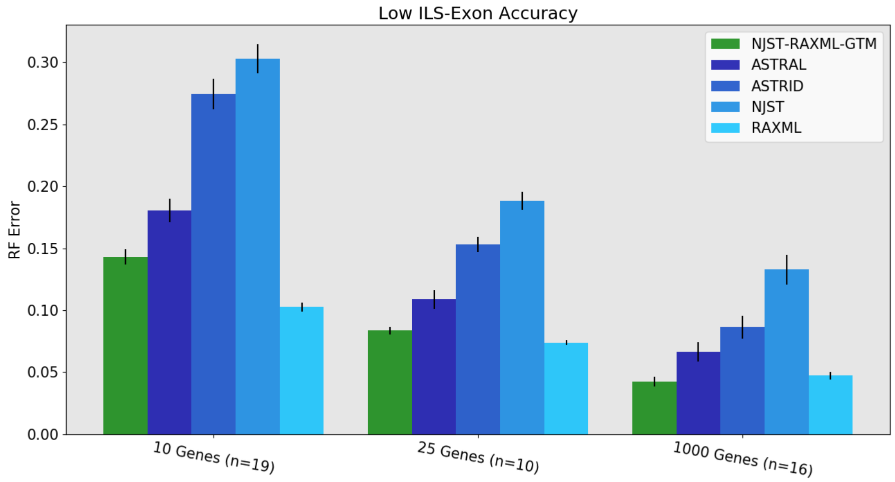

Figure S12: **Experiment 3: Comparison of NJst-RAxML-GTM to RAxML and other species tree methods on 1000-species datasets with low ILS.** The value for n is the number of replicates on which all 5 methods completed; missing replicates indicate RAxML exceeding runtime limits on 10 and 25 genes (the 16 1000-gene RAxML trees are taken from [1]). Error bars show standard error of the replicate average.

References

[1] Molloy, E.K., Warnow, T.: Statistically consistent divide-and-conquer pipelines for phylogeny estimation using NJMerge. Algorithms for Molecular Biology **14**(1), 14 (2019). doi:10.1186/s13015-019-0151-x
