# Supplementary material for: Unblended disjoint tree merging using GTM improves species tree estimation
Source: BMC Genomics. 2020 Apr 16;21(Suppl 2):235. doi: 10.1186/s12864-020-6605-1 (PMC7161100; doi:10.1186/s12864-020-6605-1)
Supplement: Supplementary file 4 — Additional file 4 Additional tables. This document provides additional tables for the performance study. [file 12864_2020_6605_MOESM4_ESM.pdf]

Additional File 4: Additional Tables

Vladimir Smirnov   Tandy Warnow

August 15, 2019

Table 1: **Number of replicates, out of 20 in each model condition, that RAxML, ASTRAL, and NJMerge complete on within the allowed 4 hour timeframe on 1000-species datasets**.

	RAxML	ASTRAL	NJMerge
1000 exons, high ILS	0	1	20
1000 introns, high ILS	0	16	20
1000 exons, low ILS	0	20	20
1000 introns, low ILS	0	20	20
25 exons, high ILS	0	20	0
25 introns, high ILS	0	20	0
25 exons, low ILS	10	20	17
25 introns, low ILS	5	20	20
10 exons, high ILS	12	13	0
10 introns, high ILS	6	19	0
10 exons, low ILS	19	20	3
10 introns, low ILS	18	20	14

Table 2: **Tree error rates for NJst-RAxML-GTM and RAxML on 1000-species datasets with low ILS.** The value for n is the number of replicates being compared, where a RAxML tree is available (finished within the time limit). The 16 exon 1000-gene RAxML trees are taken from the NJMerge study. Improved accuracy can be obtained using FastTree-RAxML-GTM.

	NJst-RAxML-GTM	RAxML
10 Exons (n=19)	0.143	0.103
10 Introns (n=18)	0.096	0.076
25 Exons (n=10)	0.083	0.074
25 Introns (n=5)	0.055	0.058
1000 Exons (n=16)	0.042	0.047
1000 Introns (n=20)	0.026	N/A

Table 3: **Tree error rates for high ILS, NJst-ASTRAL-GTM vs ASTRAL.** The value for n is the number of replicates being compared (where ASTRAL finished within the time limit). 1000 species, max subset size 120.

	NJst-ASTRAL-GTM	ASTRAL
10 Exons (n=13)	0.489	0.663
10 Introns (n=19)	0.442	0.646
25 Exons (n=20)	0.344	0.542
25 Introns (n=20)	0.289	0.354
1000 Exons (n=1)	0.062	0.067
1000 Introns (n=16)	0.057	0.059

Table 4: **Average runtime (seconds) over low ILS exons, NJst-RAxML-GTM vs RAxML on 1000-species datasets.** The value for n is the number of replicates being compared, i.e., where a RAxML tree is available. The 1000-gene RAxML trees are taken from the NJMerge study, and these are RAxML's last result on a 48-hour time limit (hence, the flat 172800 second timing for these). The RAxML constraint trees for the 1000-gene NJst-RAxML-GTM analyses were also used from the NJMerge study [1], and timings are shown for a subset of replicates that were rerun to gather timings. Finally, the 1000 FastTree2 gene trees used for NJst were reported to take 65 minutes. This is scaled to the number of genes being used.

	NJst-RAxML-GTM	RAxML
**10 Genes (n=19)**		
-Gene trees	39.0	n.a.
-NJst	7.6	n.a.
-RAxML subtrees	864.3	n.a.
-GTM	0.4	n.a.
-Total	911.3	7,313.7
**25 Genes (n=10)**		
-Gene trees	98.0	n.a.
-NJst	7.6	n.a.
-RAxML subtrees	1,504.8	n.a.
-GTM	0.4	n.a.
-Total	1,610.8	10,539.4
**1000 Genes (n=17)**		
-Gene trees	3,900.0	n.a.
-NJst	7.7	n.a.
-RAxML subtrees	69,322.4	n.a.
-GTM	0.4	n.a.
-Total	73,230.5	172,800.0

Table 5: **Average runtime (seconds) of NJst-RAxML-GTM and RAxML on 1000-species high ILS datasets with varying numbers of exons.** The value for n is the number of replicates being compared, i.e., where a RAxML tree is available. The 1000-gene RAxML trees are taken from the NJMerge study, and these are RAxML's last result on a 48-hour time limit (hence, the flat 172800 second timing for these). The RAxML constraint trees for 1000-gene NJst-RAxML-GTM were also used from the NJMerge study [1], and timings are shown for a subset of replicates that were rerun to gather timings. Finally, the 1000 FastTree2 gene trees used for NJst were reported to take 65 minutes. This is scaled to the number of genes being used.

	NJst-RAxML-GTM	RAxML
**10 Genes (n=12)**		
-Gene trees	39.0	n.a.
-NJst	7.7	n.a.
-RAxML subtrees	621.5	n.a.
-GTM	0.4	n.a.
-Total	668.6	10,135.6
**25 Genes (n=20)**		
-Gene trees	98.0	n.a.
-NJst	7.7	n.a.
-RAxML subtrees	1,478.5	n.a.
-GTM	0.4	n.a.
-Total	1,584.6	n.a.
**1000 Genes (n=20)**		
-Gene trees	3,900.0	n.a.
-NJst	8.3	n.a.
-RAxML subtrees	24,548.8	n.a.
-GTM	0.4	n.a.
-Total	28,457.5	172,800.0

Table 6: **Average runtime (seconds) over high ILS introns on 1000-species datasets for NJst-ASTRAL-GTM vs. ASTRAL.** The value for n is the number of replicates being compared, where ASTRAL trees are available. The 1000-gene ASTRAL trees are taken from the NJMerge study [1]. The 1000 FastTree2 gene trees were reported to take 65 minutes. This is scaled to the number of genes being used and applied to both methods. 1000 species, max subset size 120.

	NJst-ASTRAL-GTM	ASTRAL
**10 Genes (n=18)**		
-Gene trees	39.0	39.0
-NJst	7.7	n.a.
-ASTRAL	50.7	8,617.0
-GTM	0.4	n.a.
-Total	97.8	8,656.0
**25 Genes (n=20)**		
-Gene trees	98.0	98.0
-NJst	7.7	n.a.
-ASTRAL	69.0	5,441.4
-GTM	0.4	n.a.
-Total	175.1	5,539.4
**1000 Genes (n=16)**		
-Gene trees	3,900.0	3,900.0
-NJst	7.7	n.a.
-ASTRAL	4,048.9	149,145.9
-GTM	0.4	n.a.
-Total	7,949.3	153,045.9

References

[1] Molloy, E.K., Warnow, T.: Statistically consistent divide-and-conquer pipelines for phylogeny estimation using NJMerge. Algorithms for Molecular Biology **14**(1), 14 (2019). doi:10.1186/s13015-019-0151-x
